# Contribution of histone N-terminal tails to the structure and stability of nucleosomes^☆^^☆☆^

**DOI:** 10.1016/j.fob.2013.08.007

**Published:** 2013-08-22

**Authors:** Wakana Iwasaki, Yuta Miya, Naoki Horikoshi, Akihisa Osakabe, Hiroyuki Taguchi, Hiroaki Tachiwana, Takehiko Shibata, Wataru Kagawa, Hitoshi Kurumizaka

**Affiliations:** a Laboratory of Structural Biology, Graduate School of Advanced Science and Engineering, Waseda University, 2-2 Wakamatsu-cho, Shinjuku-ku, Tokyo 162-8480, Japan; b RIKEN, 2-1 Hirosawa, Wako-shi, Saitama 351-0198, Japan; c Program in Chemistry and Life Science, Department of Interdisciplinary Science and Engineering, School of Science and Engineering, Meisei University, 2-1-1 Hodokubo, Hino-shi, Tokyo 191-8506, Japan

**Keywords:** Histone tail, Nucleosome, Chromatin, Crystal structure, Thermal stability assay, tlH2A, human histone H2A lacking N-terminal tail, tlH2B, human histone H2B lacking N-terminal tail, tlH3, human histone H3 lacking N-terminal tail, tlH4, human histone H4 lacking N-terminal tail, wt, wild-type, SHL, superhelical location, RMSD, root mean square deviation, PDB, Protein Data Bank

## Abstract

Histones are the protein components of the nucleosome, which forms the basic architecture of eukaryotic chromatin. Histones H2A, H2B, H3, and H4 are composed of two common regions, the “histone fold” and the “histone tail”. Many efforts have been focused on the mechanisms by which the post-translational modifications of histone tails regulate the higher-order chromatin architecture. On the other hand, previous biochemical studies have suggested that histone tails also affect the structure and stability of the nucleosome core particle itself. However, the precise contributions of each histone tail are unclear. In the present study, we determined the crystal structures of four mutant nucleosomes, in which one of the four histones, H2A, H2B, H3, or H4, lacked the N-terminal tail. We found that the deletion of the H2B or H3 N-terminal tail affected histone–DNA interactions and substantially decreased nucleosome stability. These findings provide important information for understanding the complex roles of histone tails in regulating chromatin structure.

## Highlights

• The crystal structures of four N-terminal tail-less nucleosomes have been determined.
• H2B N-terminal tail deletion affected histone–DNA interactions in nucleosomes.
• H3 N-terminal tail deletion affected histone–DNA interactions in nucleosomes.
• H2B or H3 N-terminal deletion decreased the nucleosome stability.

## 1 Introduction

**Fig. 1:**
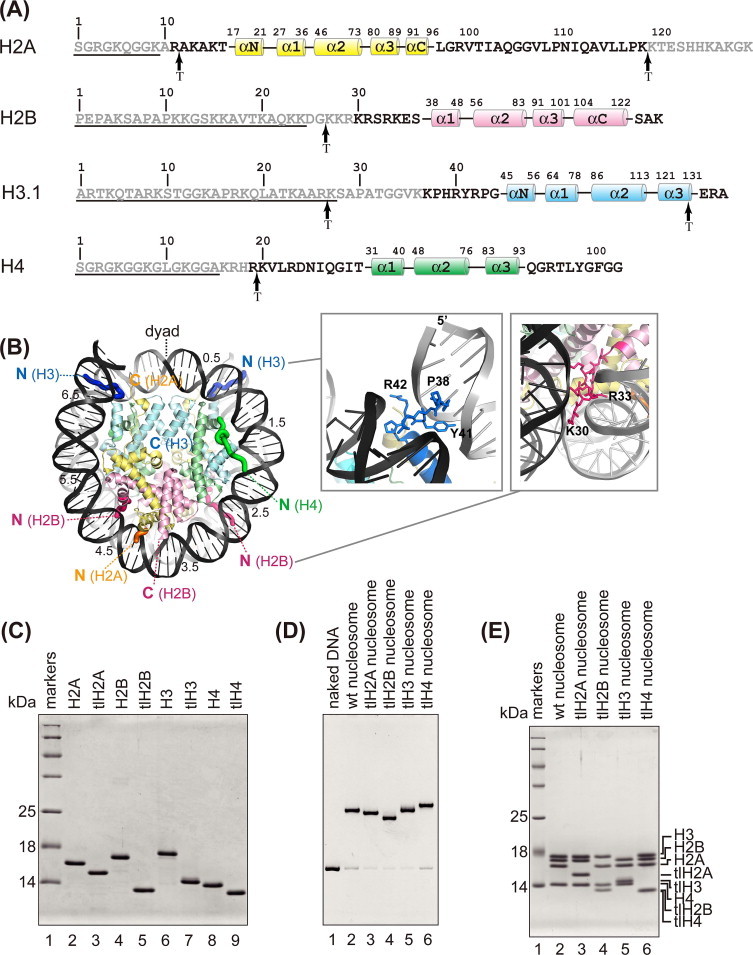
(A) The sequences of the N- and C-terminal regions and the secondary structures of histones H2A, H2B, H3, and H4. The deleted residues in histones tlH2A, tlH2B, tlH3, and tlH4 are underlined. The arrows labeled “T” mark the trypsin cleavage sites on the nucleosome [40,53]. Grey characters indicate residues that could not be modeled in the crystal structure of the wt nucleosome (PDB code 3AFA) [28,30]. (B) The locations of the N- and C-terminal regions of histones and the superhelical locations (SHL) of DNA are labeled on the crystal structure of the wt nucleosome. Histones H2A, H2B, H3 and H4 are colored yellow, pink, blue and green, respectively. Close-up views around the N-terminal regions of H3 and H2B are shown in the right panels. (C) SDS–PAGE analysis of purified intact and tailless human histones H2A, H2B, H3, and H4. (D) Reconstituted wt and tailless nucleosomes were purified by a Prep Cell apparatus and analyzed by non-denaturing 6% PAGE. (E) Analysis of the histone compositions of purified wt and tailless nucleosomes by 18% SDS–PAGE. (For interpretation of the references to color in this figure legend, the reader is referred to the web version of this article.)

The basic unit of chromatin is the nucleosome core particle, which contains 145–147 base pairs of DNA [1–3]. The DNA binds on the surface of the histone octamer, composed of two copies of the four core histones, H2A, H2B, H3, and H4. In the nucleosome structure, each core histone contains two common regions, the “histone fold” and the “histone tail”. Histone tails are flexible regions that flank both ends of the histone fold (Fig. 1(A)) [4,5]. In the nucleosome, the histone fold is responsible for the formation of stable H2A–H2B and H3–H4 dimers, and the histone octamer is composed of two H2A–H2B dimers and two H3–H4 dimers. On the other hand, the N-terminal tails of the four core histones and the C-terminal tail of histone H2A protrude from the nucleosome core particle [2]. These histone tails are known to interact with nucleosomal DNA, and the interactions are substantially retained even in the highly acetylated state [6]. In addition, the histone tails not only contact the DNA wrapped around the histone octamer, but also bind to linker DNA [7] and the acidic patches of the neighboring nucleosomes [1,2,8]. These interactions between histone tails and DNA may play critical roles in the formation of higher-order chromatin.

Biochemical studies of “tailless” nucleosomes have revealed the functional importance of histone tails. The N-terminal tails of H2A, H3, and H4 are considered to function in the formation of higher-order chromatin [8–12], and the H2B N-terminal tail is specifically required for chromosome condensation [13]. Removal of the histone tails increases the accessibility of the nucleosomal DNA, probably by enhancing the nucleosome dynamics [14–18] and/or reducing the nucleosome stability [19,20]. Consistently, deletion and alanine scanning mutation analyses of the N-terminal tail of H3 [20] or deletion of the N-terminal tail of H2B [21] revealed enhanced nucleosome sliding along the DNA. Interestingly, opposite effects have been reported for nucleosomes lacking the N-terminal tail of H2B and/or H4 [20]. A molecular dynamics simulation also suggested that the truncation of the histone tail affects the nucleosome structure [22]. The function of each histone N-terminal tail may be redundant, because the removal of the N-terminal tail of each single histone did not have an obvious effect on cell viability [11,23–25], although the simultaneous deletion of two N-terminal tails from the histone pairs H2A/H2B [23,24], H3/H4 [24], or H2A/H4 [11] compromised cell survival.

The histone tails are highly basic, and contain residues that are targets of post-translational modifications, such as acetylation, methylation, phosphorylation, and crotonylation [26,27]. These histone–tail modifications modulate the histone–DNA and nucleosome–nucleosome interactions, and may play important roles during transcription, replication, recombination, and DNA repair. Therefore, it is important to understand the molecular mechanisms by which the histone tails affect the nucleosome structure and stability.

In the present study, we determined the crystal structures of four nucleosomes lacking the N-terminal tail of one of the histones, H2A, H2B, H3, and H4. We evaluated the contribution of each histone N-terminal tail to the nucleosome stability and structure.

## 2 Materials and methods

### 2.1 Preparation and crystallization of nucleosomes containing tailless histones

Human H2A, H2B, H3.1 and H4 lacking the N-terminal tail regions were overexpressed in *Escherichia coli* with N-terminal His_6_-tags. The His_6_ tag was removed by thrombin treatment during the purification procedure. Four non-native residues, glycine–serine–histidine–methionine from the expression vector, remained at the N-terminus of each histone. Previously reported protocols were used to express, purify, and reconstitute the tailless nucleosomes [28]. A 146 bp palindromic sequence derived from human α-satellite DNA was used for nucleosome reconstitution [1,29]. Each of these tailless histones was incorporated into nucleosomes by the salt-dialysis method [28,30], and the nucleosomes were purified by native polyacrylamide gel electrophoresis [29]. Crystals for all of the tailless nucleosomes were obtained by the hanging drop vapor diffusion method, using 20 mM potassium cacodylate (pH 6.0), 50 mM KCl, and 110–155 mM MnCl_2_ as the crystallization solution. Drops composed of 1 μl nucleosome solution and 1 μl crystallization solution were equilibrated against 500 μl of reservoir solution, containing 20 mM cacodylate (pH 6.0), 35–40 mM KCl, and 60–80 mM MnCl_2_, at 20 °C.

### 2.2 Crystallographic data collection and structure determination

The diffraction data were collected at the beamline AR-NW12A at the Photon Factory (KEK, Tsukuba, Japan), at a wavelength of 1.0000 Å. Crystals were soaked in cryoprotectant solutions containing 20 mM potassium cacodylate (pH 6.0), 40 mM KCl, 60 mM MnCl_2_, 30% 2-methyl-2,4-pentanediol, and 2% trehalose, and were flash-cooled in a stream of nitrogen gas at 100 K. The data were indexed, integrated, and scaled with *HKL2000* [31], and were further processed using the *CCP4* suite programs [32]. All structures were solved by the molecular replacement method with the program *MOLREP* [33], using the structure of the intact human nucleosome (Protein Data Bank code 3AFA) as the search model. All models were checked using sigma-A-weighted composite omit maps during the modeling. The models were rebuilt with *COOT* [34] and refined with *CNS* [35]. The statistics for data collection and refinement are provided in Table 1. All molecular graphics images were generated using *PyMOL* (pymol.sourceforge.net) [36].

### 2.3 Exonuclease assay

The exonuclease assay was conducted according to the same method described previously [37]. Briefly, each reconstituted nucleosome, containing tlH2A, tlH2B, tlH3, or tlH4, was treated with 5 units of *Escherichia coli* exonuclease III (Takara), in 10 μl of 50 mM Tris–HCl (pH 8.0), 5 mM MgCl_2_, and 1 mM DTT. The reaction was continued for 0, 2, 4, or 8 min at 37 °C, and was stopped by the addition of 55 μl of proteinase K solution (20 mM Tris–HCl (pH 8.0), 20 mM EDTA, 0.5% SDS, and 0.5 mg ml^−1^ proteinase K (Roche)). After a 15 min treatment at room temperature, the DNA was extracted with phenol/chloroform, precipitated with ethanol, and dissolved in Hi-Di Formamide (Applied Biosystems). The DNA samples were then analyzed by 10% denaturing PAGE, with a gel containing 7 M urea in 0.5× TBE buffer (21 V cm^−1^ for 1.5 h).

### 2.4 Thermal stability assay

The nucleosome stability was monitored by the thermal stability assay. SYPRO Orange (SIGMA-ALDRICH) preferentially binds to hydrophobic regions of proteins, but not to nucleic acids [38]. Therefore, thermal denaturation of the nucleosome can be detected, as the fluorescence signals of the SYPRO Orange bound to the denatured histones. Each nucleosome, containing tlH2A, tlH2B, tlH3, or tlH4 (final concentration, 2.25 μM), was prepared in 20 μl of a solution composed of 18 mM Tris–HCl (pH 7.5), 0.9 mM EDTA, 0.9 mM DTT, and SYPRO Orange (final concentration, 5×). The sample temperature was increased by the StepOnePlus™ Real-Time PCR unit (Applied Biosystems), and the fluorescence signals were measured with this system. Since the wavelength at the fluorescence emission maximum of SYPRO Orange is 570 nm, the fluorescence filter ‘filter 3’, which covers the emission wavelength ranges of the TAMRA (580 nm) and NED (575 nm) dyes, was used for detecting the fluorescence of SYPRO Orange bound to the denatured histones. The temperature gradient was from 25 to 95 °C, in steps of 1 °C/min. The fluorescence intensity from the SYPRO Orange probe bound to the denatured histones was automatically converted to the normalized reporter value (Rn), and the Rn value was plotted every minute.

## 3 Results and discussion

### 3.1 Preparation of nucleosomes containing the tailless H2A, H2B, H3, or H4 histones

We bacterially expressed and purified four histone mutants (tlH2A, tlH2B, tlH3, tlH4), which lacked the N-terminal 9, 24, 27, and 15 amino acid residues of H2A, H2B, H3, and H4, respectively (Fig. 1(A) and (C)). These truncated N-terminal regions of the histones contain many previously identified post-translational modification sites [26,39]. These deletion mutants were designed based on the tailless histones obtained by the trypsin protease treatment [40], and the previous crystal structures of nucleosomes (Fig. 1(B)) [1,28,30,41].

**Fig. 2:**
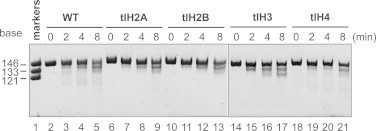
Exonuclease III digestion patterns of wt and tailless nucleosomes. Nucleosomes were digested for 0 (lanes 2, 6, 10, 14, and 18), 2 (lanes 3, 7, 11, 15, and 19), 4 (lanes 4, 8, 12, 16, and 20), or 8 (lanes 5, 9, 13, 17, and 21) min at 37 °C by *Escherichia coli* exonuclease III. The reaction was stopped by the addition of proteinase K, and the DNA was extracted with phenol/chloroform, precipitated with ethanol, and dissolved in Hi–Di Formamide. The purified DNA samples were analyzed by 10% denaturing PAGE.

We then reconstituted four nucleosomes, each containing one of the tailless histones. Reconstitution was performed by the salt dialysis method [28,30], and the nucleosomes were purified by native polyacrylamide gel electrophoresis using a Prep Cell apparatus [29]. The purified nucleosomes containing one of the tailless histones migrated differently on the native polyacrylamide gel (Fig. 1(D)), and contained stoichiometric amounts of the core histones (Fig. 1(E)). The exonuclease treatment assay revealed that all four tailless nucleosomes exhibited similar susceptibility towards ExoIII digestion, as compared to the wt nucleosome (Fig. 2). These results suggested that the DNA ends were similarly wrapped within the nucleosomes, and that tlH2A, tlH2B, tlH3, and tlH4 were properly incorporated into nucleosomes.

### 3.2 Structures of the tailless nucleosomes

**Fig. 3:**
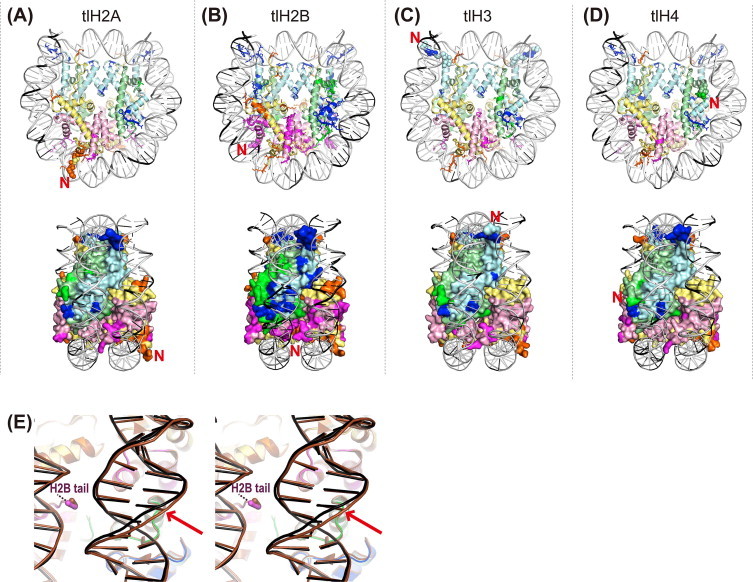
(A–D) Upper column: Crystal structures of the four tailless nucleosomes. Histone H2A is colored yellow, H2B is pink, H3 is cyan, H4 is pale green, and DNA is white. The nucleotides with RMSDs ≥1.2 Å, as compared with the structure of the wt nucleosome, are shown in black. Similarly, the histone residues with RMSDs ≥0.6 Å are highlighted in each deep color. To reduce the effects of crystal packing, the larger RMSD values between the two symmetric histones were adopted to discuss the structural alterations. The N-terminal residues without secondary structures near the truncated tails are depicted as space-filling models. Lower column: Side view. Histones are depicted in surface representations, to reveal the perturbations on the DNA-binding surface. (E) Stereo view of an example of the changes in the DNA conformation upon tail truncation: The DNA trajectories around the H2B N-terminal regions in tlH2B and wt nucleosomes are superimposed. The wt nucleosome is colored brown. The red arrow indicates a 2.03 Å shift. (For interpretation of the references to color in this figure legend, the reader is referred to the web version of this article.)

We next crystallized all four tailless nucleosomes, and determined their structures at resolutions ranging from 3.0 to 3.4 Å (Fig. 3, Table 1). All four tlH2A, tlH2B, tlH3, and tlH4 nucleosome crystals belonged to the space group *P*2_1_2_1_2_1_, which is the same as the wt nucleosome crystal (PDB code 3AFA), and the packing arrangements were almost identical among the four tailless nucleosomes. The root mean square deviations (RMSDs) between the structures of the wt nucleosome and each tailless nucleosome were calculated by the superimposition of all Cα atoms of the histones and all phosphorus (P) atoms of the DNA. The overall RMSD values for the Cα and P atoms to the wt nucleosome were 0.499 and 0.703 for tlH2A, 0.667 and 0.897 for tlH2B, 0.502 and 0.627 for tlH3, and 0.568 and 0.749 for tlH4, respectively (Table 1). Therefore, the histone structure and the DNA path in the tailless nucleosomes are not significantly different from those of the wt nucleosome. We then compared the DNA trajectory in the tlH2B nucleosome with that in the wt nucleosome (Fig. 3(E)), since the largest RMSD value for the DNA was observed between the tlH2B and wt nucleosomes (Table 1). The N-terminal tail of H2B directly binds to the DNA, and the N-terminal truncation of H2B slightly, but clearly, affected the DNA trajectory near its binding site (Fig. 3(E)).

### 3.3 The N-terminal truncation of histone H3 perturbs the histone–DNA interactions

**Fig. 4:**
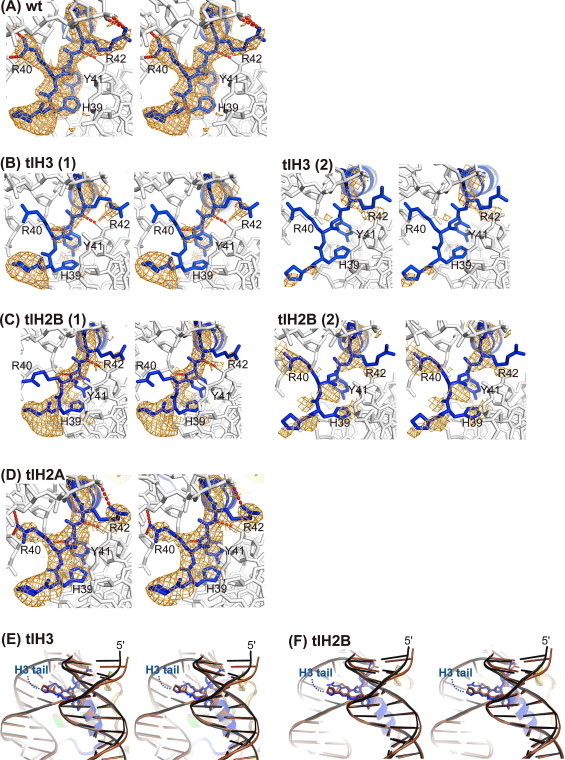
(A–D) Simulated annealing *F*_o_*− F*_c_ omit maps (2.8*σ*) for the N-terminal region of H3 in wt (A), tlH3 (B), tlH2B (C), and tlH2A (D) nucleosomes (stereo views). H3 residues 1–44 were omitted for the calculation. Hydrogen bonds between the H3 N-terminal region and the DNA are denoted by red dashed lines. Histone H3 and DNA are colored blue and white, respectively. In panels B and C, the two H3 histones are shown. (E, F) Conformational changes in the entry/exit regions of the DNA near the H3 N-terminal region, upon the removal of the H3 (E) and H2B (F) N-terminal tails (stereo views). The structure of the wt nucleosome (brown) is superimposed. (For interpretation of the references to color in this figure legend, the reader is referred to the web version of this article.)

We compared the structure and electron density maps of the tlH3 nucleosome with those of the wt nucleosome. We found that the electron density map of the H3 K37–R42 region of the tlH3 nucleosome, which is a binding site for DNA, was very ambiguous as compared to that of the wt nucleosome (Fig. 4(A) and (B)). The H3 H39–R42 region directly binds to a minor groove of the two DNA gyres in the wt nucleosome: the side chain of the H3 H39 residue is inserted in the minor groove of the entry gyre, and the H3 R40 and R42 side-chains form hydrogen bonds with the atoms of the sugar ring, the adenine base, and the phosphate backbone of another DNA gyre (Figs. 1(B) and 4(A)). In the tlH3 nucleosome, the hydrogen bonds between the DNA and the H3 R40 and R42 residues could be disrupted or weakened (Fig. 4(B)). Alternatively, the H3 K37–R42 binding to DNA may be stably retained but more variable in the tlH3 nucleosome, thus producing the ambiguous electron densities. However, previous analyses, such as single molecule transcription experiments [42], molecular dynamics simulations [22], and FRET measurements [20], indicated that the deletion of the H3 N-terminal tail enhances the transient unwrapping of DNA at the entry/exit regions. Therefore, we prefer the conclusion that some histone–DNA contacts are disrupted in the tlH3 nucleosome, thus reducing its stability.

The crystal structure of the nucleosome containing the centromeric H3 variant, CENP-A, revealed that CENP-A structurally differs from the conventional histone H3 at the N-terminal region in the nucleosome, and this CENP-A specific N-terminal structure may cause the unwrapping of DNA at the entry/exit regions in the nucleosome [37]. These facts indicated that the H3 N-terminal region contributes to the wrapping of DNA within the nucleosome. Consistently, in the wt nucleosome, the H3 N-terminal H39-R42 region penetrates into the minor groove of the DNA, and the H3 R40 and R42 side-chains directly bind to the DNA. In the present study, we found that the H39–R42 region of histone H3 becomes “less stable” and/or “poorly organized” upon the deletion of the H3 N-terminal amino acid residues 1–27. Such perturbations of the H3–DNA interactions at the N-terminal region may be partly responsible for the instability of the tlH3 nucleosome (see below).

### 3.4 The N-terminal truncation of histone H2B perturbs the histone–DNA interactions

**Fig. 5:**
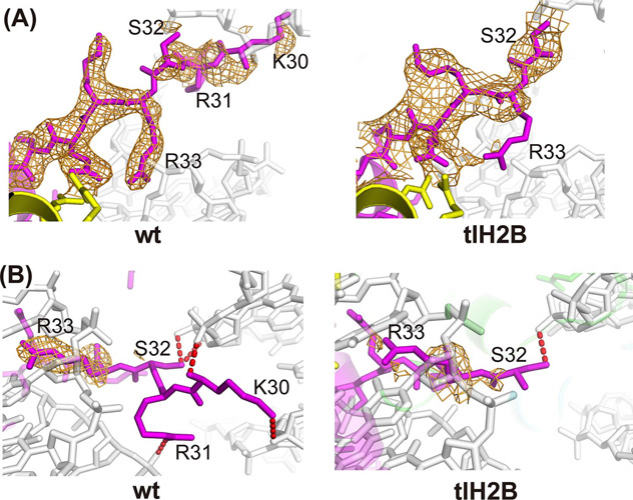
(A) The H2B N-terminal regions in wt and tlH2B nucleosomes. Histone H2B and DNA are colored magenta and white, respectively. Simulated annealing *F*_o_*− F*_c_ omit maps calculated without the H2B residues 1–37 (2.5*σ*) are shown. (B) The same region as in panel A, but in a different orientation to show the interaction with DNA. Hydrogen bonds between the H2B N-terminal region and the DNA are denoted by red dashed lines. The same omit maps as in panel A are shown only around H2B R33, for clarity. (For interpretation of the references to color in this figure legend, the reader is referred to the web version of this article.)

The H2B N-terminal tail passes between the two gyres of the DNA superhelix (Fig. 1(B)). The H2B R31 and R33 residues interact with one DNA gyre, and K30 and S32 form hydrogen bonds with the other DNA gyre in the wt nucleosome (Fig. 5) [28,30,41]. The H2B deletion of residues 1–24 affected the structure of the remaining H2B N-terminal region, in which the electron density of the region preceding the S32 residue was missing in the tlH2B nucleosome (Fig. 5(A)). Concomitantly, the H2B–DNA interactions that involve the K30 and R31 residues were disordered in the tlH2B nucleosome (Fig. 5(B)). The electron density corresponding to the side chain moiety of the H2B R33 residue was also missing in the tlH2B nucleosome (Fig. 5(A) and (B)). Therefore, the absence of the H2B–DNA interaction in the tlH2B nucleosome (K30, R31, and R33 residues) may alter the DNA trajectory near the binding site (Fig. 3(E)), and may affect the nucleosome stability. Single molecule experiments revealed three broad regions of strong histone–DNA interactions on the nucleosome core particle [43]. The strongest region is located at the dyad, and the other two strong regions are ∼ ±50 bp from the dyad. The latter regions include the interaction sites with the H2B N-terminal region, suggesting that this region contributes to enhance the affinity between the DNA and the histone octamer in nucleosomes. These findings are consistent with the previous report that the removal of the H2B N-terminal tail modulates nucleosome positioning and promotes uncatalyzed nucleosome sliding [21].

It has been proposed that the H2B N-terminal tail plays a role in the structural polymorphism of nucleosomes through DNA untwisting, which could modify the interactions between the distal H3 N-terminal tail and the DNA at the entry-exit site [44]. Consistently, the electron density around the H3 H39–R42 region was ambiguous in the tlH2B nucleosome structure, as compared to the wt nucleosome (Fig. 4(A) and (C)), despite the facts that the full-length H3 was reconstituted in the tlH2B nucleosome, and the H2B N-terminal tails were located far away from those of H3 (Fig. 1(B)). This may not be the only reason for the low-resolution structure, because the electron density of the corresponding region was clearly observed in the tlH2A nucleosome structure at similar resolution (Fig. 4(D)). Actually, the conformations of the DNA entry/exit regions interacting with the H3 N-terminal regions were influenced similarly in both the tlH2B and tlH3 nucleosomes (Fig. 4(E) and (F)). Altogether, the removal of the H2B N-terminal tail seems to destabilize the extensive interactions between the histone and DNA.

### 3.5 The N-terminal truncations of histones H2A and H4

**Fig. 6:**
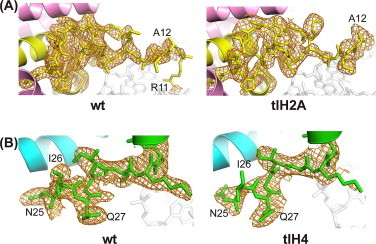
Comparison of the N-terminal regions of wt nucleosome with tlH2A (A) and tlH4 (B) nucleosomes. Simulated annealing *F*_o_*− F*_c_ omit maps were calculated for residues 1–28 of H2A and 1–32 of H4, respectively, and contoured at the 2.5*σ* level. Histones H2A, H2B, H3, H4 and DNA are colored yellow, pink, cyan, green, and semi-transparent white, respectively. (For interpretation of the references to color in this figure legend, the reader is referred to the web version of this article.)

The N-terminal tail of histone H2A is exposed on the disk face of the nucleosome, and is close to the H2B C-terminal tail (Fig. 1(B)). The deletion of the H2A residues 1–9 did not affect the overall nucleosome structure (Fig. 3(A)) and the remaining N-terminal tail structure (Fig. 6(A)). Thus, we concluded that the impact on the nucleosome structure upon the truncation of the H2A N-terminal residues 1–9 is minimal. Similarly, the H4 deletion of residues 1–15 also did not significantly affect the overall and remaining N-terminal tail structures in the nucleosome (Fig. 6(B)). The H4 N-terminal tail directly binds to the acidic patch of the neighboring nucleosome surface [1,2,8,45], and is reportedly important for the compact folding of chromatin fibers [46,47]. The H4 1–19 region, especially H4 residues 14–19, has been demonstrated to be essential for chromatin fiber compaction, but the N-terminal tails of H2A, H2B, and H3 were not fully required for the compaction [47]. In the present structure of the tlH4 nucleosome that lacks H4 residues 1–15, no significant structural differences were found in the remaining N-terminal regions of both H4 histones (Fig. 6(B)), or in the crystal packing contacts with the H2A acidic patch. This is consistent with the previous report that the deletion of the H4 residues 1–13 did not influence array compaction [47]. Therefore, the H4 residues 16–19 may function in chromatin fiber compaction.

### 3.6 The N-terminal tails of H2B and H3 contribute to nucleosome stability

**Fig. 7:**
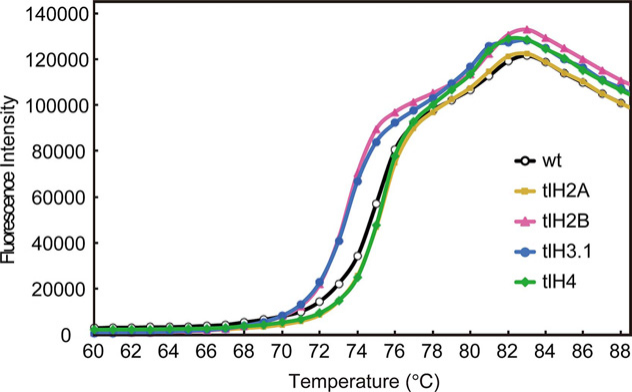
Effect of the removal of the histone N-terminal tails on the thermal stabilities of the nucleosomes. Each nucleosome, containing tlH2A, tlH2B, tlH3, or tlH4 (final concentration, 2.25 μM), was mixed with SYPRO Orange, and the sample temperature was increased by the StepOnePlus™ Real-Time PCR unit (Applied Biosystems). The temperature gradient was from 25 to 95 °C, in steps of 1 °C/min. Fluorescence signals of SYPRO Orange bound to the denatured histones were measured, and the thermal denaturing curves of the wt, tlH2A, tlH2B, tlH3, and tlH4 nucleosomes are shown in black, yellow, pink, blue, and green, respectively. (For interpretation of the references to color in this figure legend, the reader is referred to the web version of this article.)

Altered stability in the proteolytically generated tailless nucleosomes was observed by the thermal denaturation assay, but not by the salt dissociation assay [19]. Therefore, we performed a thermal stability assay with nucleosomes containing tlH2A, tlH2B, tlH3, or tlH4, in the presence of SYPRO Orange. SYPRO Orange is a dye that preferentially binds to hydrophobic regions of proteins [38]. Therefore, the binding of SYPRO Orange to native, folded proteins is low, whereas the binding increases when proteins are denatured and the internal hydrophobic regions become exposed to the solvent. In fact, small amounts of SYPRO Orange bound to histones that were properly incorporated into nucleosomes. In contrast, the amount of SYPRO Orange bound to histones drastically increased when the histones were denatured, as a consequence of the thermal disruption of nucleosomes (Fig. 7).

Under the experimental conditions used in this study, the wt nucleosome was disrupted in a two-step manner (with a biphasic curve), which probably reflects the ordered disassembly of H2A–H2B, followed by H3–H4 (Fig. 7) [48]. Ausio et al. previously reported that the wt nucleosome exhibited biphasic denaturation, and the first (74.5 °C) and second (∼80 °C) melting transitions [19] are roughly in accordance with our thermal denaturation profile (Fig. 7). In the nucleosomes containing tlH2A and tlH4, the initial phase of the disruption occurred at slightly higher temperatures than that of the wt nucleosome (Fig. 7), suggesting that the nucleosome stability was not affected by the deletion of H2A residues 1–9 or H4 residues 1–15. Since the H4 residues 16–19, which may be important for chromatin fiber compaction [47], were still retained in the tlH4 nucleosome, further deletion of H4 N-terminal residues may affect the nucleosome stability. By contrast, in nucleosomes containing tlH2B (lacking residues 1–24) and tlH3 (lacking residues 1–27), the initial phase of disruption clearly occurred at lower temperatures, as compared to the wt nucleosome (Fig. 7). These results suggested that the N-terminal tails of H2B (1–24) and H3 (1–27) make important contributions towards the stability of nucleosomes. The tlH2B nucleosome instability revealed by the thermal stability assay is consistent with the previous observation that the deletion of the H2B 3–22 region confers a Sin (SWI/SNF-independent) phenotype [49], which accompanies nucleosome instability [50–52]. The deletion of the H3 N-terminal tail reportedly destabilizes the H2A–H2B dimer within the nucleosome [20]. This is consistent with our observation that the first melting transition of the tlH3 nucleosome (Fig. 7) probably reflects the H2A–H2B disassembly occurring at a lower temperature than that in the wt nucleosome.

These thermal stability data may be explained by our crystal structures of the tlH2B and tlH3 nucleosomes. The histone–DNA contacts were weakened in the tlH2B and tlH3 nucleosomes (Figs. 4 and 5). In addition, alanine point mutations of the H3 H39, R40, and R42 residues reportedly increase nucleosome mobility [20]. These results suggested that the interactions of these residues with DNA may be important for the stable wrapping of the DNA around the histone octamer.

Coordinates and structure factors have been deposited in the Protein Data Bank (tlH2A nucleosome, PDB: 3W96; tlH2B nucleosome, PDB: 3W97; tlH3 nucleosome, PDB: 3W98; and tlH4 nucleosome, PDB: 3W99).

## Figures and Tables

**Table 1:** Data collection and refinement statistics.

Crystal data	tlH2A	tlH2B	tlH3	tlH4
*Data collection statistics*				
Space group^a^	*P*2_1_2_1_2_1_	*P*2_1_2_1_2_1_	*P*2_1_2_1_2_1_	*P*2_1_2_1_2_1_
*a* (Å)	104.5	105.8	104.8	105.7
*b* (Å)	109.3	109.7	109.3	109.3
*c* (Å)	175.7	175.4	176.2	175.7
Resolution range (Å)	50.0–3.00	50.0–3.20	50.0–3.40	50.0–3.00
No. of reflections	242705	243734	140869	298483
No. of unique reflections	40008	33745	27657	41127
Completeness (%) b	98.0 (98.5)	97.4 (50.9)	98.9 (100)	99.3 (99.9)
*R*_sym_ (%) b^,^^c^	7.2 (71.1)	9.5 (56.1)	10.4 (56.3)	9.0 (71.8)
*I*/*σ* (I) b	12.7 (2.2)	9.5 (3.1)	7.6 (3.0)	10.9 (2.9)
*Refinement statistics*				
*R*_work_ (%) d/*R*_free_ (%)	24.8/29.6	26.9/32.1	26.0/30.3	24.4/31.3
No. of protein residues	749	752	752	752
No. of base pairs of DNA	145	146	145	145
No. of ions	3	1	1	1
No. of water molecules	0	0	0	0
RMSD from ideal				
Bond length (Å)	0.009	0.008	0.006	0.005
Bond angles (°)	1.31	1.27	1.15	1.06
Average B-factors (Å^2^)				
Protein	86.5	90.6	99.9	89.5
DNA	149.7	154.7	162.9	152.3
Ion	111.8	76.3	80.1	75.1
PDB code	3W96	3W97	3W98	3W99
*RMSD from wild type*				
Cα (histones) (Å)	0.499	0.667	0.502	0.568
P (DNA) (Å)	0.703	0.897	0.627	0.749

a The crystal of the wild-type nucleosome (PDB 3AFA) belongs to the space group *P*2_1_2_1_2_1_, with unit cell dimensions *a* = 105.8, *b* = 109.5, *c*= 180.9 Å.

b Values in parentheses are for the highest-resolution shell.

c *R*_sym_ = Σ*_hkl_*Σ_*i*_|*I*_*hkl*, *i*_  – *<I_hkl_>*|/Σ_*hkl*_Σ_*i*_*I*_*hkl*, *i*_.

d *R*_work_ = Σ*_hkl_*∥*F*_o_| – |*F*_c_∥/Σ_*hkl*_|*F*_o_|. *R*_free_ is calculated with 5% of the total reflections held aside throughout the refinement.
