# Corrigendum to: Contribution of histone N‐terminal tails to the structure and stability of nucleosomes

**DOI:** 10.1002/2211-5463.12508

**Published:** 2018-08-23

**Authors:** Wakana Iwasaki, Yuta Miya, Naoki Horikoshi, Akihisa Osakabe, Hiroyuki Taguchi, Hiroaki Tachiwana, Takehiko Shibata, Wataru Kagawa, Hitoshi Kurumizaka

Recently, Iwasaki *et al*. 1 noticed that the tlH4 (tail‐less H4) protein used in their study contained an additional amino acid sequence, MVDLQAAANSLVIHM, at its N‐terminal end. This sequence, which is derived from the cloning vector, was mistakenly inserted. Specifically, the tlH4 protein contains this additional sequence downstream of the N‐terminal thrombin cleavage site and before the H4K16 residue, as shown below.

GSH‐MVDLQAAANSLVIHM‐KRHRKVLRDNIQGITKPAIRRLARRGGVKRISGLIYEETRGVLKVFLENVIRDAVTYTEHAKRKTVTAMDVVYALKRQGRTLYGFGG

The inserted sequence is not seen in the electron density maps (PDB: http://www.rcsb.org/pdb/search/structidSearch.do?structureId=3w99), but neither was the authentic N‐terminal tail of histone H4 in the other three related structures (PDB: http://www.rcsb.org/pdb/search/structidSearch.do?structureId=3w96, http://www.rcsb.org/pdb/search/structidSearch.do?structureId=3w97, and http://www.rcsb.org/pdb/search/structidSearch.do?structureId=3w98) analyzed in this paper. Thus, the additional sequence did not influence the structural results, even though it may be questionable for the interpretation of the biological properties of the supposedly ‘tail‐less’ histone H4. The authors apologize for any inconvenience this may have caused, and would like to have the opportunity to make this correction.
